# Recent Advances in Translational Adipose-Derived Stem Cell Biology

**DOI:** 10.3390/biom11111660

**Published:** 2021-11-09

**Authors:** Darius Widera

**Affiliations:** Stem Cell Biology and Regenerative Medicine Group, School of Pharmacy, University of Reading, Reading RG6 6UB, UK; d.widera@reading.ac.uk; Tel.: +44-(0)-118-378-7053

Multipotent mesenchymal stem cells/marrow stromal cells (MSCs), originally discovered in the bone marrow by Alexander Friedenstein as early as 1968 [1] and further characterised in 1991 by Arnold I. Caplan [2], are multipotent adult stem cells present in many adult tissues. MSCs have been shown to differentiate into several mesenchymal derivatives including adipogenic, chondrogenic, and osteogenic cells whilst also possessing a considerable regenerative potential mediated by paracrine factors [3]. In 2001, the presence of MSC-like cells was reported in human fat tissue [4]. Since then, several independent studies have validated this initial report and referred to this multipotent cell population as adipose-derived stem cells (ASCs), adipose-derived adult stem cells, adipose-derived adult stromal cells, adipose-derived stromal cells, adipose stromal cells, adipose mesenchymal stem cells, lipoblast, pericyte, preadipocyte, or processed lipoaspirate cells. It is not surprising that this ambiguous, incoherent nomenclature has caused substantial confusion in the field. To address this problem, the International Fat Applied Technology Society (IFAT) suggested at its annual meeting in 2004 to adopt the term ASCs.

Two decades after their initial discovery, ASCs have become a clinical reality and are considered an alternative to MSCs, with over 400 clinical trials registered on the clinicaltrial.gov database (accessed on 3 November 2021). ASCs possess a differentiation capacity similar to that of MSCs from other sources and exhibit paracrine ‘bystander’ effects in many degenerative conditions. Their clear advantage over other sources of MSCs is the relative abundance of the source material, which can be obtained in ample amounts using minimally invasive surgical procedures.

However, their high therapeutic promise, the ease of their isolation, and over-enthusiastic reporting by the media has resulted in a considerable increase in unlicensed direct-to-consumer businesses, which exploit regulatory loopholes to offer therapeutic ASCs with little scientific evidence and no therapeutic value.

This Special Issue invited original research articles and reviews focused on recent evidence-based advances in ASC biology as well as their translational application.

In their comprehensive review, Ong et al. discussed the heterogeneity of human ACSs, which can potentially represent an obstacle in the translation of basic ACS research into the clinics [5]. Among other factors, the source of ASCs (e.g., subcutaneous vs. visceral fat), donor and inter-subject variations as well as details of the study design were identified as potential hurdles for translational use of ASCs. Importantly, this heterogeneity might affect not only the differentiation spectrum of ASCs but also their paracrine regenerative potential. The paracrine potential of ASCs is key for developing cell-free therapies based on the use of ASC secretomes and is reviewed in this Special Issue by Trzyna and Banas-Zabczyk [6]. This review summarises the current knowledge on soluble factors (growth factors, cytokines, and chemokines) and provides an overview of the cargo of extracellular vesicles (miRNAs, mRNA, and proteins) released by ASCs. Importantly, a synergistic action of soluble factors and EV cargo were shown to be responsible for the paracrine, regeneration-modulating action of ASCs [7]. Thus, detailed information on the composition of the ASC secretome is key for understanding the mode of action in ASC transplantation.

Secretion of paracrine neurotrophic and pro-angiogenic factors as paracrine factors has been identified as one of the modes of action in ASC-mediated improvement of symptoms in experimental spinal cord injury and related in vitro models [8]. In the study by Delfi and colleagues, the authors compared the neurotrophic and angiogenic activity of human and canine ASCs [9]. Interestingly, the secretome released by ASCs from both species mediated similar neurotrophic and pro-angiogenic effects. These findings might be of particular relevance for the development of large animal models of secretome-mediated regeneration, since small animal models only poorly reflect regenerative processes in humans.

If addition to their paracrine activity, ASCs mediate regeneration via direct differentiation if the organ or tissue affected by degeneration is mesodermal (e.g., bone, fat tissue or cartilage). In this context, Kladnicka et al. [10] demonstrated that chronic exposure of ASCs to the environmental pollutant 2,2-bis (4-chlorophenyl)-1,1-dichlorethylenedichlorodiphenyldichloroethylene (p,p’-DDE) modifies mitochondrial respiration during adipogenic differentiation. Since this phenomenon could bias ASCs towards adipogenesis, these results provide a potential link between environmental pollutants produced during the Anthropocene epoch and the current obesity epidemic especially prevalent in developed and developing countries.

In order to obtain clinically relevant cell numbers, ASCs must be expanded in vitro over several passages. However, extensive in vitro expansion of stem cells has been linked with chromosomal instability and a reduction in differentiation capacity [11,12]. This can partly be attributed to a phenotypic change after removal of stem cells from their three-dimensional (3D) niche, resulting in forced apical basal polarity [13]. Modern 3D cell culture techniques offer the advantage of mimicking the stem cell niche more closely than traditional cultivation on flat surfaces whilst often preserving the in vivo phenotype after isolation of stem cells from their niche. In this Special Issue, O’Donnell et al. reported that human subcutaneous ASCs embedded in 3D methacrylated gelatine hydrogels can be efficiently expanded in 3D-printed bioreactors [14]. Moreover, they demonstrated that this does not affect the differentiation potential of ASCs. In another study within this issue, Bicer et al. demonstrated that human lipoaspirate ASCs can be efficiently expanded within 3D nanofibrillar cellulose hydrogels [8]. Consistent with the findings reported by O’Donnell, 3D cell culture did not interfere with the differentiation potential of ASCs. Importantly, electrical stimulation of ASCs within the 3D scaffold resulted in a significantly increased osteogenic potential compared with both ASCs in 2D and cells in 3D without exposure to electrical stimuli.

Differentiation of ASCs has been suggested to correlate with defined patterns of intracellular calcium oscillation. In their study, Torre at al. showed that undifferentiated ASCs and cells that differentiate towards the osteogenic and adipogenic fates are characterised by very distinct calcium oscillation patterns [15]. Notably, this methodology can be exploited as a non-invasive technique for assessing ASC differentiation in vitro.

Overall, the field of ASC biology is moving rapidly towards clinical translation. However, despite the promise of ASC-based therapies targeting previously incurable disorders and conditions, all attempts at the translational application of ASCs must be based on evidence and not driven by enthusiasm combined with commercial interests. Specifically, ASC-based therapies should, even in cases of compassionate use, meet the criteria of no toxicity and tumorigenicity and have higher efficacy compared with the placebo in a relevant animal model. In addition, the mode of action must be fully characterised. Only if this is ensured and the direct-to-consumer market is tightly regulated by authorities can a real benefit to patients and society be achieved without damaging the credibility of the field.

## Data Availability

Not applicable.
